# The crystal structures of four *N*-(4-halophen­yl)-4-oxo-4*H*-chromene-3-carboxamides

**DOI:** 10.1107/S2056989014027054

**Published:** 2015-01-01

**Authors:** Ligia R. Gomes, John Nicolson Low, Fernando Cagide, Fernanda Borges

**Affiliations:** a FP-ENAS-Faculdade de Ciências de Saúde, Escola Superior de Saúde da UFP, Universidade Fernando Pessoa, Rua Carlos da Maia, 296, P-4200-150 Porto, Portugal; b Department of Chemistry, University of Aberdeen, Meston Walk, Old Aberdeen, AB24 3UE, Scotland; c CIQ/Departamento de Quιmica e Bioquιmica, Faculdade de Ciências, Universidade do Porto, 4169-007 Porto, Portugal

**Keywords:** crystal structure, drug design, chromones, conformation, supra­molecular structure

## Abstract

Four *N*-(4-halophen­yl)-4-oxo-4*H*-chromene-3-carboxamides (halo = F, Cl, Br and I), *N*-(4-fluoro­phen­yl)-4-oxo-4*H*-chromene-3-carboxamide, C_16_H_10_FNO_3_, *N*-(4-chloro­phen­yl)-4-oxo-4*H*-chromene-3-carboxamide, C_16_H_10_ClNO_3_, *N*-(4-bromo­phen­yl)-4-oxo-4*H*-chromene-3-carboxamide, C_16_H_10_BrNO_3_, *N*-(4-iodo­phen­yl)-4-oxo-4*H*-chromene-3-carboxamide, C_16_H_10_INO_3_, have been structurally characterized. The mol­ecules are essentially planar and each exhibits an *anti* conformation with respect to the C—N rotamer of the amide and a *cis* geometry with respect to the relative positions of the C_arom_—C_arom_ bond of the chromone ring and the carbonyl group of the amide. The structures each exhibit an intra­molecular hydrogen-bonding network comprising an N—H⋯O hydrogen bond between the amide N atom and the O atom of the carbonyl group of the pyrone ring, forming an *S*(6) ring, and a weak C_arom_—H⋯O inter­action with the O atom of the carbonyl group of the amide as acceptor, which forms another *S*(6) ring. All four compounds have the same supra­molecular structure, consisting of *R*_2_^2^(13) rings that are propagated along the *a-*axis direction by unit translation. There is π–π stacking involving inversion-related mol­ecules in each structure.

## Abstract 2

In four *N*-(4-halophen­yl)-4-oxo-4*H*-chromene-3-carboxamides, halo = -F, -Cl, -Br and -I, the mol­ecules are essentially planar and exhibit *anti* conformations with respect to the C—N rotamer of the amide and with *cis* geometries with respect to the relative positions of the C3_arom_—C2_arom_ bond of the chromone ring and the carbonyl group of the amide.

## Chemical context   

Chromones are a group of natural and synthetic oxygen heterocyclic compounds having a high degree of chemical diversity that is frequently linked to a broad array of biological activities (Gaspar *et al.* 2014 ▸). Parkinson’s disease (PD) is a degenerative disorder of the central nervous system with an aetiology not yet completely clarified. There is no cure for PD, but medications, surgery and multidisciplinary management can provide relief from the symptoms. PD seems to be associated with a decrease in central levels of dopamine triggered by oxidative stress. These processes, among other factors, are mediated by the isoform B of the mono­amino oxidase (MAO-B). Hence, the search for novel agents that can selectively inhibit MAO-B is of paramount relevance. In this context, the decoration of chromone, a privileged structure for the discovery and development of new chemical entities (NCEs), have led to the preparation of chromone carboxamides and to promising outcomes since preliminary data indicate that chromone-3-carboxamides are selective MAO-B inhibitors (Gaspar, Reis *et al.*, 2011 ▸; Gaspar, Silva *et al.*, 2011 ▸).

Previous results showed that the carbonyl group of the chromone moiety and the amide function play an important role in the establishment of hydrogen inter­actions with the MAO-B active pocket. In addition, the presence of a phenyl substituent attached to the amide seems to play a pivotal role in the potency conveyed by the ligand (Helguera *et al.*, 2013 ▸). In this context, some *N*-(4-halophen­yl)-4-oxo-4*H*-chromene-3-carboxamides (1)–(4), shown in the scheme, have been synthesized and structurally characterized in order to rationalize the structural factors that may affect the selectivity and the potency of their inhibitory activities towards MAO-B. These structures are compared with *N*-(4-phen­yl)-4-oxo-4*H*-chromene-2-carboxamide and *N*-(4-bromo­phen­yl)-4-oxo-4*H*-chromene-2-carboxamide, compounds (5) and (6) (Reis *et al.*, 2013 ▸; Gomes *et al.*, 2013 ▸), which do not show inhibitory activities against human MAO-B.
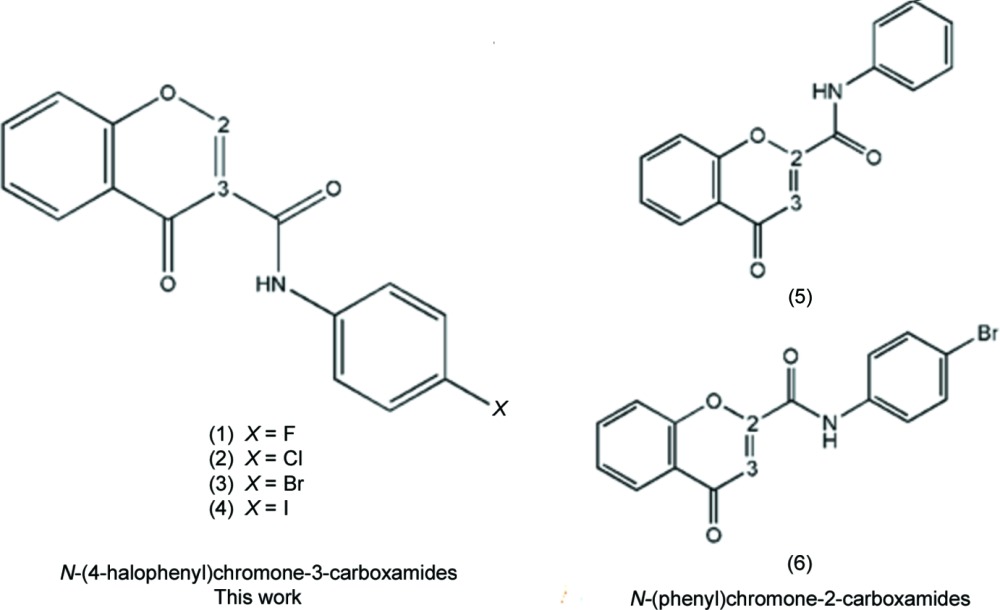


## Structural commentary   

**Figure 1:**
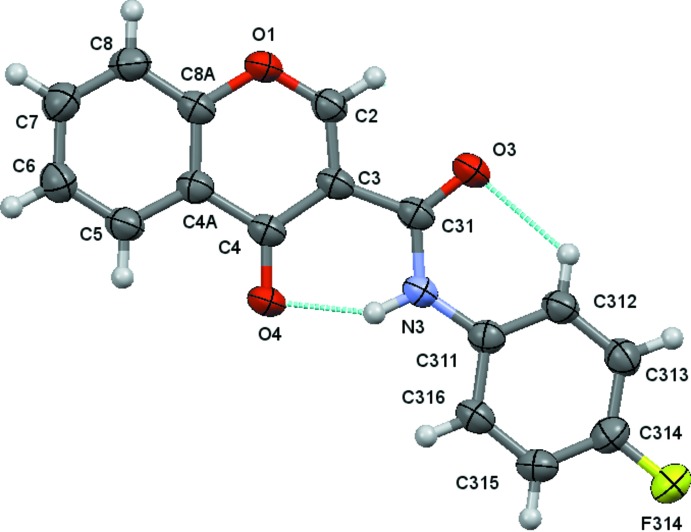
A view of the asymmetric unit of (1), showing the atom-numbering scheme. Displacement ellipsoids are drawn at the 80% probability level. Dashed lines indicate the intra­molecular contacts.

**Figure 2:**
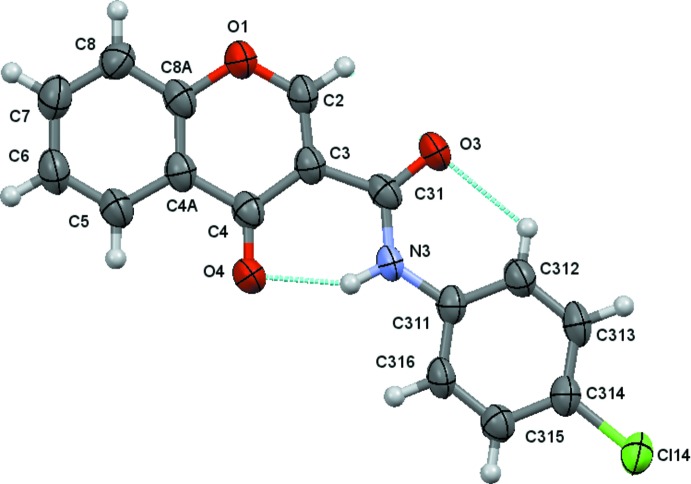
A view of the asymmetric unit of (2), showing the atom-numbering scheme. Displacement ellipsoids are drawn at the 80% probability level. Dashed lines indicate the intra­molecular contacts.

**Figure 3:**
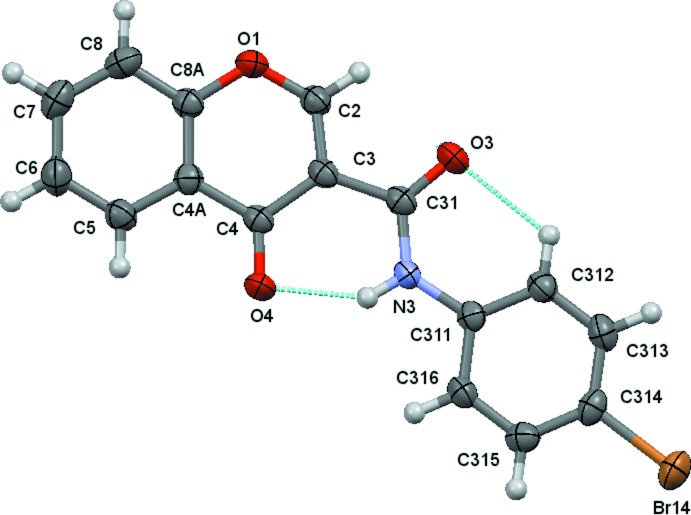
A view of the asymmetric unit of (3), showing the atom-numbering scheme. Displacement ellipsoids are drawn at the 80% probability level. Dashed lines indicate the intra­molecular contacts.

**Figure 4:**
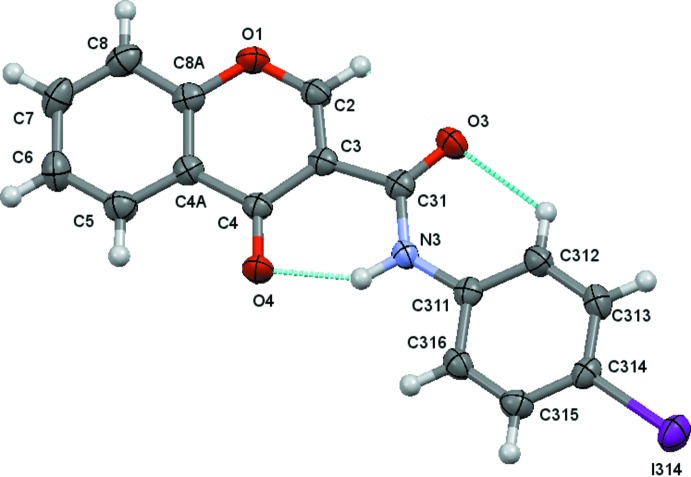
A view of the asymmetric unit of (4), showing the atom-numbering scheme. Displacement ellipsoids are drawn at the 80% probability level. Dashed lines indicate the intra­molecular contacts.

The structural analysis of (1)–(4) confirmed them to be *N*-(4-halophen­yl)-4-oxo-4*H*-chromene-3-carboxamides with halosubstituents F (Fig. 1 ▸), Cl (Fig. 2 ▸), Br (Fig. 3 ▸) and I (Fig. 4 ▸), respectively, as depicted in the scheme. Figs. 1 ▸–4 ▸ ▸ ▸ show the displacement ellipsoid diagrams with the adopted labelling schemes. All compounds crystallize in the space group *P* 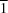. Compounds (1) and (2) are isostructural, as are compounds (3) and (4). The cell lengths are very similar in each pair of compounds.

The title compounds display similar structures, which are reflected in the mol­ecular geometries and conformations; the values of the dihedral angles between the mean planes of the chromone ring and the exocyclic phenyl ring of the *N*-phenyl-4-oxo-4*H*-chromene-3-carboxamides are close to 2° in the case of the F, Cl pair [2.51 (3) and 1.95 (7)°, respectively,] and close to 5° for the Br, I pair [4.90 (10) and 5.37 (10)°, respectively]. In *N*-phenyl-4-oxo-4*H*-chromene-2-carboxamide (5) (Reis *et al.*, 2013 ▸), the dihedral angle between the mean planes of the chromone ring and the phenyl ring is 6.57° and in *N*-(4-bromo­phen­yl)-4-oxo-4*H*-chromene-2-carboxamide (6), the structural isomer of (3) (Gomes *et al.*, 2013 ▸), the dihedral angle between the mean planes of the chromone ring and the phenyl ring is 5.0 (2)°. Selected dihedral angles are given in Table 1 ▸.

In (1) and (2), the maximum deviations from the mean plane of the 10 atoms of the chromone ring plus the three carboxamide atoms O3, C31 and N3, are 0.1220 (8) and 0.1319 (17) Å, respectively, both for atom O3 (r.m.s. deviations of fitted atoms = 0.0519 and 0.0571 Å, respectively). In (3) and (4), the deviations of O3 from the mean plane defined above are 0.0384 (14) and 0.0342 (15) Å, respectively (r.m.s. deviations of fitted atoms = 0.0314 Å in both compounds). In the case of (3) and (4), atom C2 shows the greatest deviation from the mean plane having deviations of 0.0569 (18) and 0.0596 (18) Å, respectively. These values indicate that the carboxamide groups are practically planar with the chromone ring, particularly in the case of the Br and I chromone carboxamide derivatives. This planarity may be related to the inter­nal hydrogen-bond pattern in those mol­ecules, which thus defines the mol­ecular conformations.

**Figure 5:**
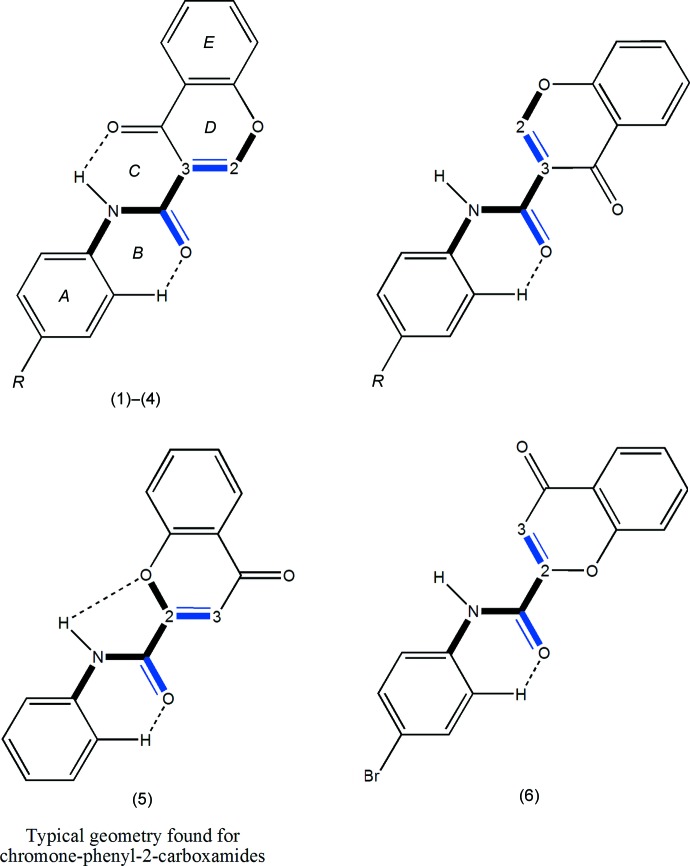
*Anti*-rotamer conformations around the C—N rotamer for the 3-carboxamides (top) and for the 2-carboxamide isomers (bottom), showing the relative positions of the C3_arom_—C2_arom_ bond of the chromone ring with respect to the carb­oxy­lic group of the amide: *cis* (right) or *trans* (left) geometries.

The conformational features herein established are probably most relevant for the extrapolation of the inhibitory MAO-B activities of chromone carboxamides as they are related to the inter­molecular forces responsible for enzyme–ligand binding affinity. The data can explain the MAO-B selectivity found for chromone-3-carboxamides (1)–(4), as opposed to the lack of activity presented by chromone-2-carboxamides (5) and (6). As seen in the scheme, (1)–(4) are *N*-(phen­yl)-4-oxo-4*H*-chromene-**3**-carboxamides while (5) and (6) are *N*-(phen­yl)-4-oxo-4*H*-chromene-**2**-carboxamides. As can be seen in Fig. 5 ▸, an *anti* conformation is adopted with respect to the C—N rotamer of the amide in all of the compounds. Nevertheless, due to the asymmetry of the chromone residue, the *anti* conformation can assume a *cis* (*a*) or *trans* (*b*) geometry with respect to the relative position of the carbonyl O atom of the carboxamide and the C2_arom_—C3_arom_ bond of the chromone. Compounds (1)–(4) exhibit a *cis* relation between these bonds, as can be seen in the ellipsoid diagrams, Figs. 1 ▸–4 ▸ ▸ ▸. This mol­ecular conformation permits the formation of two intra­molecular hydrogen bonds, which generate a network that probably enhances their planarity. Details of the intra­molecular hydrogen-bonding inter­actions are given in Tables 2 ▸ ▸ ▸ to 5 ▸. Specifically for each mol­ecule, there is an intra­molecular N—H⋯O hydrogen bond between the amide nitro­gen and the oxygen atom of the carbonyl group, O4, of the chromone ring, forming an *S*(6) ring identified as ring *C*. In addition, the carbonyl oxygen of the amide, O3, acts as the acceptor for a weak inter­action with an *ortho* hydrogen of the exocyclic phenyl ring, forming another S(6) ring, *B*. The corresponding *trans* structures (top right in Fig. 5 ▸) would probably only allow the formation of a weak hydrogen-bonding inter­action with an *ortho* hydrogen atom of the exocyclic phenyl ring. It is inter­esting to compare the inter­nal hydrogen-bonding network presented by the title compounds with those of the analogous 4-oxo-*N*-(substituted phen­yl)-4*H*-chromene-2-carboxamides (Reis *et al.*, 2013 ▸) and (Gomes *et al.*, 2013 ▸), compounds (5) and (6). Previous studies concerning the structures of the chromone-2-carboxamides show that the majority have geometries similar to compound (5), *e.g.* as in (1)–(4), they assume a *cis* conformation, but this is not the case for (6), the bromo isomer of (3), as shown in Fig. 5 ▸ (bottom right). In spite of this, none of this type of derivative displays inhibitory activity towards the MAO-B isoenzyme. When the geometries of the relative positions of rings *D* and *E* of the chromone residue with respect to rings *A* and *B* are compared, it can be seen that the effect of the 2/3 positional isomerism is to ‘reflect’ their relative positions while the effect of the *cis*/*trans* conformations is a ‘twofold rotation’ of the rings around the C_amide_— C_chromone_ bond. Those particular differences in conformation may condition the ability for docking when pharmacological activities are considered.

## Supra­molecular features   

**Figure 6:**
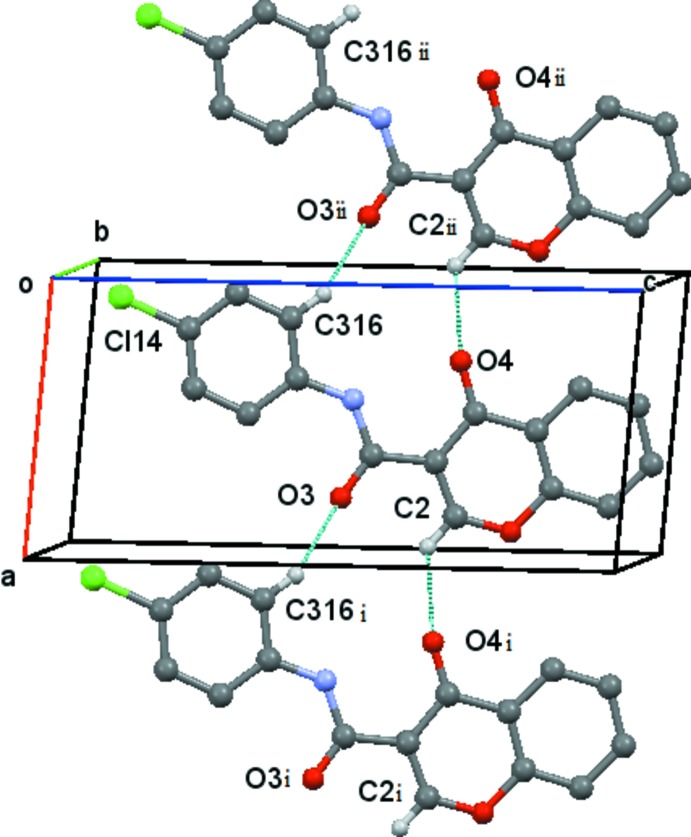
The distorted ladder formed by linked 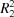
(13) rings in compound (3). The chain runs parallel to the *a* axis. Hydrogen bonds are indicated by blue dashed lines. Hydrogen atoms not involved in the hydrogen bonding have been omitted for clarity. A similar structure is found for compound (1) and all the halo-substituted compounds. [Symmetry codes: (i) *x* + 1, *y*, *z*; (ii) *x* − 1, *y*, *x*.]

Inter­molecular hydrogen-bonding information is given in Table 2 ▸ to 5. All compounds have the same supra­molecular structure in which the C2—H2⋯O4(*x* + 1, *y*, *z*) and C316—H316⋯O3(*x* − 1, *y*, *z*) form 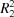
(13) ring structures, which are propagated along the *a*-axis direction by unit translation. Fig. 6 ▸ shows the Cl compound, (3), as an example.

There is π–π stacking in each compound, involving inversion-related mol­ecules in all compounds, Table 6 ▸.

## Synthesis and crystallization   

The title compounds were obtained by synthetic strategies described elsewhere (Cagide *et al.*, 2011 ▸). Chromone-3-carboxamides were synthesized using chromone-3-carb­oxy­lic acid as starting material which, after *in situ* activation with phospho­rus(V) oxychloride (POCl_3_) in di­methyl­formamide, react with the different haloanilines. Recrystallization from di­chloro­methane afforded colourless plates whose dimensions are given in Table 7 ▸.

## Refinement   

Crystal data, data collection and structure refinement details are summarized in Table 7 ▸. Amino H atoms were located in difference Fourier maps and were refined isotropically. All other H atoms were treated as riding atoms with C—H(aromatic) = 0.95 Å, *U*_iso_= 1.2*U*eq(C).

Compounds (1) and (2), reduced cell: [*a* = 6.6325 (12), *b* = 0.0577 (12), *c* = 14.671 (3) Å, α = 76.464 (7), β = 89.714 (6), γ = 74.411 (7)°, V = 641.9 (2) Å^3^], have different reduced cells in which the *x* and *z* coordinates are comparable and the *y* coordinate of (2) is close to 1 − *y* of (1). For ease of comparison of the structures of (1) and (2), the refinement reported here was carried out for the non-reduced cell of (2) in which the α and γ angles were given the supplementary values of those of the reduced unit cell. The coordinates of (1) were used as starting values and the transformation matrix for the reduced to non-reduced cell was 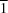 0 0 0 1 0 0 0 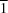. This gave the same final refinement values as those for the refinement with the reduced cell. Compounds (1) and (2) are therefore isostructural.

## Supplementary Material

Crystal structure: contains datablock(s) 1, 2, 3, 4, global. DOI: 10.1107/S2056989014027054/lh5743sup1.cif

Structure factors: contains datablock(s) 1. DOI: 10.1107/S2056989014027054/lh57431sup2.hkl

Structure factors: contains datablock(s) 2. DOI: 10.1107/S2056989014027054/lh57432sup3.hkl

Structure factors: contains datablock(s) 3. DOI: 10.1107/S2056989014027054/lh57433sup4.hkl

Structure factors: contains datablock(s) 4. DOI: 10.1107/S2056989014027054/lh57434sup5.hkl

Supporting information file. DOI: 10.1107/S2056989014027054/lh57431sup6.cml

Supporting information file. DOI: 10.1107/S2056989014027054/lh57432sup7.cml

Supporting information file. DOI: 10.1107/S2056989014027054/lh57433sup8.cml

Supporting information file. DOI: 10.1107/S2056989014027054/lh57434sup9.cml

CCDC references: 1038511, 1038510, 1038509, 1038508

Additional supporting information:  crystallographic information; 3D view; checkCIF report

## Figures and Tables

**Table 1:** Selected dihedral angles () _1_ is the dihedral angle between the mean planes of the chromene and phenyl rings and the phenyl ring. _2_ is the dihedral angle between the mean plane of the chromone ring and the plane defined by atoms O2, C31 and N3. _3_ is the dihedral angle between the mean planes of the phenyl ring and the plane defined by atoms O3, C31 and N3.

Compound	_1_	_2_	_3_
(1)	2.51(3)	5.51(12)	5.05(13)
(2)	1.95(7)	5.7(3)	4.4(3)
(3)	4.90(10)	2.0(4)	2.9(4)
(4)	5.37(10)	1.8(4)	3.6(4)

**Table 2:** Hydrogen-bond geometry (, ) for (1)

*D*H*A*	*D*H	H*A*	*D* *A*	*D*H*A*
N3H3O4	0.896(17)	1.901(17)	2.7024(13)	147.9(15)
C312H312O3	0.95	2.26	2.8714(15)	122
C2H2O4^i^	0.95	2.45	3.1645(14)	132
C316H316O3^ii^	0.95	2.46	3.3160(14)	149

Symmetry codes: (i) 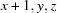; (ii) 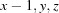.

**Table 3:** Hydrogen-bond geometry (, ) for (2)

*D*H*A*	*D*H	H*A*	*D* *A*	*D*H*A*
N3H3O4	0.85(3)	1.92(3)	2.680(3)	148(3)
C312H312O3	0.95	2.29	2.892(3)	121
C2H2O4^i^	0.95	2.47	3.194(3)	133
C316H316O3^ii^	0.95	2.45	3.286(3)	146

Symmetry codes: (i) 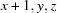; (ii) 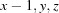.

**Table 4:** Hydrogen-bond geometry (, ) for (3)

*D*H*A*	*D*H	H*A*	*D* *A*	*D*H*A*
N3H3O4	0.86(2)	1.95(2)	2.695(2)	145(2)
C312H312O3	0.95	2.26	2.877(2)	129
C2H2O4^i^	0.95	2.41	3.167(2)	137
C316H316O3^ii^	0.95	2.47	3.314(2)	148

Symmetry codes: (i) 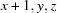; (ii) 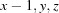.

**Table 5:** Hydrogen-bond geometry (, ) for (4)

*D*H*A*	*D*H	H*A*	*D* *A*	*D*H*A*
N3H3O4	0.92(2)	1.89(2)	2.6977(19)	145(2)
C2H2O3	0.95	2.33	2.718(2)	104
C312H312O3	0.95	2.27	2.881(2)	122
C2H2O4^i^	0.95	2.44	3.185(2)	136
C316H316O3^ii^	0.95	2.49	3.312(2)	145

Symmetry codes: (i) 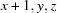; (ii) 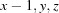.

**Table 6:** stacking (, ) *Cg*1, *Cg*2, *Cg*3 and *Cg*7 [compound (6)] are the centroids of the rings containing atoms O1, C5, C311 and C211 [compound (6)], respectively. In contacts indicated *, the planes involved are inclined to each other, the perpendicular distance between the planes is an average value and the angle between the planes is given in place of a slippage. Only interplanar interactions with *Cg* *Cg* distances 4.0 and with angles between the planes of 10 are included.

Compound	contact	distance	perp. dist.	angle between planes
(1)	*Cg*1*Cg*3^iii^	3.5187(8)	3.3226*	1.77(6)*
	*Cg*1*Cg*3^iv^	3.543(8)	3.3719*	1.77(6)*
(2)	*Cg*1*Cg*3^v^	3.5341(17)	3.3573*	0.77(13)*
	*Cg*2*Cg*3^vi^	3.6691(17)	3.3985*	3.14(13)*
(3)	*Cg*1*Cg*3^v^	3.5464(11)	3.3342*	4.66(9)*
(4)	*Cg*1*Cg*3^iii^	3.5721(11)	3.3518*	5.37(9)

Symmetry codes: (iii) *x*+1, *y*+1, *z*+1; (iv) *x*, *y*+2, *z*; (v) *x*+1, *y*, *z*+1; (vi) *x*, *y*, *z*.

**Table 7:** Experimental details

	(1)	(2)	(3)	(4)
Crystal data				
Chemical formula	C_16_H_10_FNO_3_	C_16_H_10_ClNO_3_	C_16_H_10_BrNO_3_	C_16_H_10_INO_3_
*M*_r_	283.25	299.70	344.16	391.15
Crystal system, space group	Triclinic, *P* 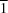	Triclinic, *P* 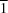	Triclinic, *P* 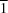	Triclinic, *P* 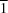
Temperature (K)	100	100	120	120
*a*, *b*, *c* ()	6.6213(5), 7.0517(5), 14.0864(10)	6.6325(12), 7.0577(12), 14.671(3)	6.6505(5), 9.3580(7), 11.0060(8)	6.6750(5), 9.4166(7), 11.2673(8)
, , ()	101.957(7), 90.047(6), 106.657(7)	103.536(7), 89.714(6), 105.589(7)	100.280(6), 90.461(6), 100.884(6)	100.974(6), 90.769(6), 100.062(6)
*V* (^3^)	615.17(8)	641.9(2)	661.24(9)	683.77(9)
*Z*	2	2	2	2
Radiation type	Mo *K*	Mo *K*	Mo *K*	Mo *K*
(mm^1^)	0.12	0.31	3.12	2.35
Crystal size (mm)	0.46 0.32 0.02	0.17 0.17 0.04	0.58 0.18 0.06	0.46 0.22 0.05

Data collection				
Diffractometer	Rigaku Saturn724+	Rigaku AFC12	Rigaku R-AXIS conversion	Rigaku R-AXIS conversion
Absorption correction	Multi-scan (*CrystalClear-SM Expert*; Rigaku, 2012 ▸)	Multi-scan (*CrystalClear-SM Expert*; Rigaku, 2012 ▸)	Multi-scan (*CrystalClear-SM Expert*; Rigaku, 2012 ▸)	Multi-scan (*CrystalClear-SM Expert*; Rigaku, 2012 ▸)
*T*_min_, *T*_max_	0.949, 0.998	0.950, 0.988	0.265, 0.835	0.411, 0.892
No. of measured, independent and observed [*I* > 2(*I*)] reflections	8176, 2789, 2393	7435, 2265, 1668	9930, 3017, 2525	10032, 3095, 2819
*R*_int_	0.056	0.078	0.045	0.026
(sin /)_max_ (^1^)	0.649	0.598	0.649	0.649

Refinement				
*R*[*F* ^2^ > 2(*F* ^2^)], *wR*(*F* ^2^), *S*	0.044, 0.135, 1.06	0.056, 0.145, 0.99	0.027, 0.058, 0.94	0.018, 0.044, 1.03
No. of reflections	2789	2265	3017	3095
No. of parameters	194	194	194	194
H-atom treatment	H atoms treated by a mixture of independent and constrained refinement	H atoms treated by a mixture of independent and constrained refinement	H atoms treated by a mixture of independent and constrained refinement	H atoms treated by a mixture of independent and constrained refinement
_max_, _min_ (e ^3^)	0.41, 0.27	0.30, 0.65	0.53, 0.69	0.67, 0.32

Computer programs: *CrystalClear-SM Expert* (Rigaku, 2012 ▸), *SHELXS97* and *SHELXL2014* (Sheldrick, 2008 ▸), *PLATON* (Spek, 2009 ▸) *Flipper 25* (Oszlnyi St, 2004 ▸), *OSCAIL* (McArdle *et al.*, 2004 ▸), *ShelXle* (Hbschle *et al.*, 2011 ▸), *Mercury* (Macrae *et al.*, 2006 ▸) and *OSCAIL* (McArdle *et al.*, 2004 ▸).

## supplementary crystallographic information

### Crystal data

**Table:**

C_16_H_10_INO_3_	*Z* = 2
*M**_r_* = 391.15	*F*(000) = 380
Triclinic, *P*1	*D*_x_ = 1.900 Mg m^−^^3^
*a* = 6.6750 (5) Å	Mo *K*α radiation, λ = 0.71075 Å
*b* = 9.4166 (7) Å	Cell parameters from 9236 reflections
*c* = 11.2673 (8) Å	θ = 1.8–27.5°
α = 100.974 (6)°	µ = 2.35 mm^−^^1^
β = 90.769 (6)°	*T* = 120 K
γ = 100.062 (6)°	Plate, colourless
*V* = 683.77 (9) Å^3^	0.46 × 0.22 × 0.05 mm

### Data collection

**Table:**

Rigaku RAXIS conversion diffractometer	3095 independent reflections
Radiation source: Sealed Tube	2819 reflections with *I* > 2σ(*I*)
Graphite Monochromator monochromator	*R*_int_ = 0.026
Detector resolution: 10.0000 pixels mm^-1^	θ_max_ = 27.5°, θ_min_ = 2.2°
profile data from ω–scans	*h* = −7→8
Absorption correction: multi-scan (*CrystalClear-SM Expert*; Rigaku, 20112)	*k* = −12→11
*T*_min_ = 0.411, *T*_max_ = 0.892	*l* = −14→14
10032 measured reflections	

### Refinement

**Table:**

Refinement on *F*^2^	0 restraints
Least-squares matrix: full	Hydrogen site location: mixed
*R*[*F*^2^ > 2σ(*F*^2^)] = 0.018	H atoms treated by a mixture of independent and constrained refinement
*wR*(*F*^2^) = 0.044	*w* = 1/[σ^2^(*F*_o_^2^) + (0.026*P*)^2^] where *P* = (*F*_o_^2^ + 2*F*_c_^2^)/3
*S* = 1.03	(Δ/σ)_max_ = 0.002
3095 reflections	Δρ_max_ = 0.67 e Å^−^^3^
194 parameters	Δρ_min_ = −0.32 e Å^−^^3^

### Special details

**Table:**

Geometry. All e.s.d.'s (except the e.s.d. in the dihedral angle between two l.s. planes) are estimated using the full covariance matrix. The cell e.s.d.'s are taken into account individually in the estimation of e.s.d.'s in distances, angles and torsion angles; correlations between e.s.d.'s in cell parameters are only used when they are defined by crystal symmetry. An approximate (isotropic) treatment of cell e.s.d.'s is used for estimating e.s.d.'s involving l.s. planes.

### Fractional atomic coordinates and isotropic or equivalent isotropic displacement parameters (Å^2^)

**Table:**

	*x*	*y*	*z*	*U*_iso_*/*U*_eq_	
I314	0.09431 (2)	0.28556 (2)	0.97093 (2)	0.02278 (5)	
O1	0.88205 (19)	0.94124 (14)	0.35573 (11)	0.0188 (3)	
O3	0.7838 (2)	0.68549 (15)	0.60794 (12)	0.0219 (3)	
O4	0.28854 (19)	0.79085 (14)	0.42255 (11)	0.0199 (3)	
N3	0.4362 (2)	0.65097 (16)	0.58280 (13)	0.0152 (3)	
H3	0.337 (3)	0.678 (2)	0.5384 (19)	0.017 (5)*	
C2	0.8340 (3)	0.85191 (19)	0.43445 (15)	0.0166 (3)	
H2	0.9434	0.8230	0.4731	0.020*	
C3	0.6432 (3)	0.79929 (17)	0.46363 (14)	0.0144 (3)	
C4	0.4692 (3)	0.83443 (18)	0.40253 (14)	0.0144 (3)	
C4A	0.5235 (3)	0.92875 (18)	0.31366 (14)	0.0149 (3)	
C5	0.3733 (3)	0.9701 (2)	0.24576 (16)	0.0193 (4)	
H5	0.2334	0.9352	0.2557	0.023*	
C6	0.4280 (3)	1.0611 (2)	0.16480 (16)	0.0221 (4)	
H6	0.3257	1.0865	0.1176	0.026*	
C7	0.6338 (3)	1.1164 (2)	0.15168 (16)	0.0217 (4)	
H7	0.6696	1.1811	0.0971	0.026*	
C8	0.7853 (3)	1.0777 (2)	0.21751 (16)	0.0211 (4)	
H8	0.9249	1.1154	0.2094	0.025*	
C8A	0.7265 (3)	0.98182 (19)	0.29605 (15)	0.0167 (3)	
C311	0.3707 (3)	0.56515 (18)	0.66915 (14)	0.0147 (3)	
C312	0.5034 (3)	0.52301 (19)	0.74728 (15)	0.0170 (3)	
H312	0.6466	0.5501	0.7426	0.020*	
C313	0.4242 (3)	0.4409 (2)	0.83219 (15)	0.0183 (3)	
H313	0.5134	0.4119	0.8857	0.022*	
C314	0.2148 (3)	0.40150 (18)	0.83828 (15)	0.0167 (3)	
C315	0.0823 (3)	0.44141 (19)	0.75977 (15)	0.0178 (3)	
H315	−0.0608	0.4127	0.7637	0.021*	
C316	0.1603 (3)	0.52325 (19)	0.67578 (15)	0.0171 (3)	
H316	0.0702	0.5512	0.6221	0.020*	
C31	0.6294 (3)	0.70661 (18)	0.55846 (15)	0.0154 (3)	

### Atomic displacement parameters (Å^2^)

**Table:**

	*U*^11^	*U*^22^	*U*^33^	*U*^12^	*U*^13^	*U*^23^
I314	0.02679 (8)	0.02415 (7)	0.01880 (6)	0.00303 (5)	0.00371 (4)	0.00890 (4)
O1	0.0120 (6)	0.0209 (6)	0.0242 (6)	0.0014 (5)	0.0022 (5)	0.0074 (5)
O3	0.0143 (6)	0.0284 (7)	0.0258 (7)	0.0053 (5)	−0.0013 (5)	0.0108 (5)
O4	0.0116 (6)	0.0256 (7)	0.0248 (6)	0.0024 (5)	0.0016 (5)	0.0116 (5)
N3	0.0136 (7)	0.0169 (7)	0.0164 (7)	0.0040 (6)	−0.0002 (5)	0.0057 (5)
C2	0.0139 (8)	0.0165 (8)	0.0192 (8)	0.0035 (6)	−0.0004 (6)	0.0027 (6)
C3	0.0138 (8)	0.0129 (8)	0.0158 (7)	0.0027 (6)	0.0005 (6)	0.0005 (6)
C4	0.0132 (8)	0.0146 (8)	0.0151 (7)	0.0027 (6)	0.0006 (6)	0.0018 (6)
C4A	0.0157 (9)	0.0141 (8)	0.0146 (7)	0.0031 (6)	0.0021 (6)	0.0017 (6)
C5	0.0169 (9)	0.0217 (9)	0.0195 (8)	0.0036 (7)	0.0019 (6)	0.0045 (6)
C6	0.0260 (10)	0.0246 (9)	0.0186 (8)	0.0088 (8)	0.0007 (7)	0.0078 (7)
C7	0.0283 (10)	0.0197 (9)	0.0198 (8)	0.0066 (7)	0.0080 (7)	0.0078 (6)
C8	0.0202 (10)	0.0193 (9)	0.0236 (9)	0.0024 (7)	0.0066 (7)	0.0047 (7)
C8A	0.0161 (9)	0.0155 (8)	0.0181 (8)	0.0035 (6)	0.0026 (6)	0.0016 (6)
C311	0.0166 (9)	0.0127 (8)	0.0145 (7)	0.0034 (6)	0.0011 (6)	0.0014 (6)
C312	0.0147 (9)	0.0183 (8)	0.0186 (8)	0.0041 (7)	−0.0002 (6)	0.0038 (6)
C313	0.0199 (9)	0.0198 (8)	0.0164 (8)	0.0065 (7)	−0.0023 (6)	0.0038 (6)
C314	0.0203 (9)	0.0148 (8)	0.0149 (7)	0.0018 (7)	0.0026 (6)	0.0039 (6)
C315	0.0148 (9)	0.0199 (9)	0.0183 (8)	0.0030 (7)	0.0018 (6)	0.0029 (6)
C316	0.0164 (9)	0.0171 (8)	0.0184 (8)	0.0055 (7)	−0.0014 (6)	0.0027 (6)
C31	0.0161 (9)	0.0140 (8)	0.0157 (7)	0.0036 (6)	0.0007 (6)	0.0010 (6)

### Geometric parameters (Å, º)

**Table:**

I314—C314	2.1023 (17)	C6—C7	1.401 (3)
O1—C2	1.340 (2)	C6—H6	0.9500
O1—C8A	1.376 (2)	C7—C8	1.385 (3)
O3—C31	1.229 (2)	C7—H7	0.9500
O4—C4	1.243 (2)	C8—C8A	1.394 (2)
N3—C31	1.357 (2)	C8—H8	0.9500
N3—C311	1.406 (2)	C311—C312	1.397 (2)
N3—H3	0.92 (2)	C311—C316	1.398 (3)
C2—C3	1.352 (2)	C312—C313	1.395 (3)
C2—H2	0.9500	C312—H312	0.9500
C3—C4	1.460 (2)	C313—C314	1.388 (3)
C3—C31	1.497 (2)	C313—H313	0.9500
C4—C4A	1.469 (2)	C314—C315	1.389 (2)
C4A—C8A	1.390 (2)	C315—C316	1.383 (2)
C4A—C5	1.404 (2)	C315—H315	0.9500
C5—C6	1.377 (3)	C316—H316	0.9500
C5—H5	0.9500		
			
C2—O1—C8A	118.48 (14)	C7—C8—H8	121.0
C31—N3—C311	128.55 (15)	C8A—C8—H8	121.0
C31—N3—H3	114.0 (15)	O1—C8A—C4A	121.30 (15)
C311—N3—H3	117.4 (15)	O1—C8A—C8	116.04 (16)
O1—C2—C3	125.61 (16)	C4A—C8A—C8	122.66 (17)
O1—C2—H2	117.2	C312—C311—C316	119.80 (16)
C3—C2—H2	117.2	C312—C311—N3	123.60 (16)
C2—C3—C4	119.41 (15)	C316—C311—N3	116.59 (15)
C2—C3—C31	115.53 (15)	C313—C312—C311	119.53 (17)
C4—C3—C31	125.07 (15)	C313—C312—H312	120.2
O4—C4—C3	124.22 (16)	C311—C312—H312	120.2
O4—C4—C4A	121.28 (16)	C314—C313—C312	119.87 (16)
C3—C4—C4A	114.49 (15)	C314—C313—H313	120.1
C8A—C4A—C5	118.05 (16)	C312—C313—H313	120.1
C8A—C4A—C4	120.59 (16)	C313—C314—C315	120.84 (16)
C5—C4A—C4	121.35 (16)	C313—C314—I314	120.05 (13)
C6—C5—C4A	120.26 (18)	C315—C314—I314	119.08 (13)
C6—C5—H5	119.9	C316—C315—C314	119.42 (17)
C4A—C5—H5	119.9	C316—C315—H315	120.3
C5—C6—C7	120.37 (18)	C314—C315—H315	120.3
C5—C6—H6	119.8	C315—C316—C311	120.53 (16)
C7—C6—H6	119.8	C315—C316—H316	119.7
C8—C7—C6	120.65 (17)	C311—C316—H316	119.7
C8—C7—H7	119.7	O3—C31—N3	124.75 (16)
C6—C7—H7	119.7	O3—C31—C3	121.00 (16)
C7—C8—C8A	117.95 (17)	N3—C31—C3	114.25 (15)
			
C8A—O1—C2—C3	−1.7 (2)	C4—C4A—C8A—C8	−176.55 (16)
O1—C2—C3—C4	3.0 (3)	C7—C8—C8A—O1	177.28 (15)
O1—C2—C3—C31	−177.33 (15)	C7—C8—C8A—C4A	−2.4 (3)
C2—C3—C4—O4	179.64 (16)	C31—N3—C311—C312	0.3 (3)
C31—C3—C4—O4	0.0 (3)	C31—N3—C311—C316	179.48 (16)
C2—C3—C4—C4A	−0.9 (2)	C316—C311—C312—C313	−0.7 (2)
C31—C3—C4—C4A	179.46 (15)	N3—C311—C312—C313	178.37 (16)
O4—C4—C4A—C8A	177.21 (15)	C311—C312—C313—C314	0.1 (3)
C3—C4—C4A—C8A	−2.3 (2)	C312—C313—C314—C315	0.8 (3)
O4—C4—C4A—C5	−1.7 (3)	C312—C313—C314—I314	−177.36 (13)
C3—C4—C4A—C5	178.81 (15)	C313—C314—C315—C316	−1.0 (3)
C8A—C4A—C5—C6	−0.2 (3)	I314—C314—C315—C316	177.17 (12)
C4—C4A—C5—C6	178.65 (16)	C314—C315—C316—C311	0.3 (3)
C4A—C5—C6—C7	−1.7 (3)	C312—C311—C316—C315	0.5 (3)
C5—C6—C7—C8	1.6 (3)	N3—C311—C316—C315	−178.64 (15)
C6—C7—C8—C8A	0.4 (3)	C311—N3—C31—O3	2.5 (3)
C2—O1—C8A—C4A	−1.8 (2)	C311—N3—C31—C3	−177.41 (15)
C2—O1—C8A—C8	178.47 (15)	C2—C3—C31—O3	2.3 (2)
C5—C4A—C8A—O1	−177.34 (15)	C4—C3—C31—O3	−178.01 (15)
C4—C4A—C8A—O1	3.7 (2)	C2—C3—C31—N3	−177.74 (14)
C5—C4A—C8A—C8	2.4 (3)	C4—C3—C31—N3	1.9 (2)

### Hydrogen-bond geometry (Å, º)

**Table:**

*D*—H···*A*	*D*—H	H···*A*	*D*···*A*	*D*—H···*A*
N3—H3···O4	0.92 (2)	1.89 (2)	2.6977 (19)	145 (2)
C2—H2···O3	0.95	2.33	2.718 (2)	104
C312—H312···O3	0.95	2.27	2.881 (2)	122
C2—H2···O4^i^	0.95	2.44	3.185 (2)	136
C316—H316···O3^ii^	0.95	2.49	3.312 (2)	145

Symmetry codes: (i) *x*+1, *y*, *z*; (ii) *x*−1, *y*, *z*.
